# De novo design of picomolar SARS-CoV-2 miniprotein inhibitors

**DOI:** 10.1126/science.abd9909

**Published:** 2020-09-09

**Authors:** Longxing Cao, Inna Goreshnik, Brian Coventry, James Brett Case, Lauren Miller, Lisa Kozodoy, Rita E. Chen, Lauren Carter, Alexandra C. Walls, Young-Jun Park, Eva-Maria Strauch, Lance Stewart, Michael S. Diamond, David Veesler, David Baker

**Affiliations:** 1 Department of Biochemistry, University of Washington, Seattle, WA 98195, USA.; 2 Institute for Protein Design, University of Washington, Seattle, WA 98195, USA.; 3 Molecular Engineering Graduate Program, University of Washington, Seattle, WA 98195, USA.; 4 Department of Medicine, Washington University School of Medicine, St. Louis, MO 63110, USA.; 5 Department of Pathology and Immunology, Washington University School of Medicine, St. Louis, MO 63110, USA.; 6 Department of Pharmaceutical and Biomedical Sciences, University of Georgia, Athens, GA 30602, USA.; 7 The Andrew M. and Jane M. Bursky Center for Human Immunology and Immunotherapy Programs, Washington University School of Medicine, St. Louis, MO 63110, USA.; 8 Howard Hughes Medical Institute, University of Washington, Seattle, WA 98195, USA.

## Abstract

Targeting the interaction between the severe acute respiratory syndrome coronavirus 2 (SARS-CoV-2) spike protein and the human angiotensin-converting enzyme 2 (ACE2) receptor is a promising therapeutic strategy. We designed inhibitors using two de novo design approaches. Computer-generated scaffolds were either built around an ACE2 helix that interacts with the spike receptor binding domain (RBD) or docked against the RBD to identify new binding modes, and their amino acid sequences were designed to optimize target binding, folding, and stability. Ten designs bound the RBD, with affinities ranging from 100 picomolar to 10 nanomolar, and blocked SARS-CoV-2 infection of Vero E6 cells with median inhibitory concentration (IC_50_) values between 24 picomolar and 35 nanomolar. The most potent, with new binding modes, are 56- and 64-residue proteins (IC_50_ ~ 0.16 nanograms per milliliter). Cryo–electron microscopy structures of these minibinders in complex with the SARS-CoV-2 spike ectodomain trimer with all three RBDs bound are nearly identical to the computational models. These hyperstable minibinders provide starting points for SARS-CoV-2 therapeutics.

## Miniproteins against SARS-CoV-2

Severe acute respiratory syndrome coronavirus 2 (SARS-CoV-2) is decorated with spikes, and viral entry into cells is initiated when these spikes bind to the host angiotensin-converting enzyme 2 (ACE2) receptor. Many monoclonal antibody therapies in development target the spike proteins. Cao *et al.* designed small, stable proteins that bind tightly to the spike and block it from binding to ACE2. The best designs bind with very high affinity and prevent SARS-CoV-2 infection of mammalian Vero E6 cells. Cryo–electron microscopy shows that the structures of the two most potent inhibitors are nearly identical to the computational models. Unlike antibodies, the miniproteins do not require expression in mammalian cells, and their small size and high stability may allow formulation for direct delivery to the nasal or respiratory system.

*Science*, this issue p. 426

## Teaser

Designed miniproteins bind tightly to the SARS-CoV-2 spike protein and prevent binding to the host cell receptor.

Severe acute respiratory syndrome coronavirus 2 (SARS-CoV-2) infection generally begins in the nasal cavity, with virus replicating there for several days before spreading to the lower respiratory tract (*1*). Delivery of a high concentration of a viral inhibitor into the nose and into the respiratory system generally might therefore provide prophylactic protection and/or therapeutic benefit for treatment of early infection and could be particularly useful for healthcare workers and others coming into frequent contact with infected individuals. A number of monoclonal antibodies are in development as systemic treatments for coronavirus disease 2019 (COVID-19) (*2*–*6*), but these proteins are not ideal for intranasal delivery because antibodies are large and often not extremely stable molecules, and the density of binding sites is low (two per 150 KDa antibody); antibody-dependent disease enhancement (*7*–*9*) is also a potential issue. High-affinity spike protein binders that block the interaction with the human cellular receptor angiotensin-converting enzyme 2 (ACE2) (*10*) with enhanced stability and smaller sizes to maximize the density of inhibitory domains could have advantages over antibodies for direct delivery into the respiratory system through intranasal administration, nebulization, or dry powder aerosol. We found previously that intranasal delivery of small proteins designed to bind tightly to the influenza hemagglutinin can provide both prophylactic and therapeutic protection in rodent models of lethal influenza infection (*11*).

## Design strategy

**Fig. 1:**
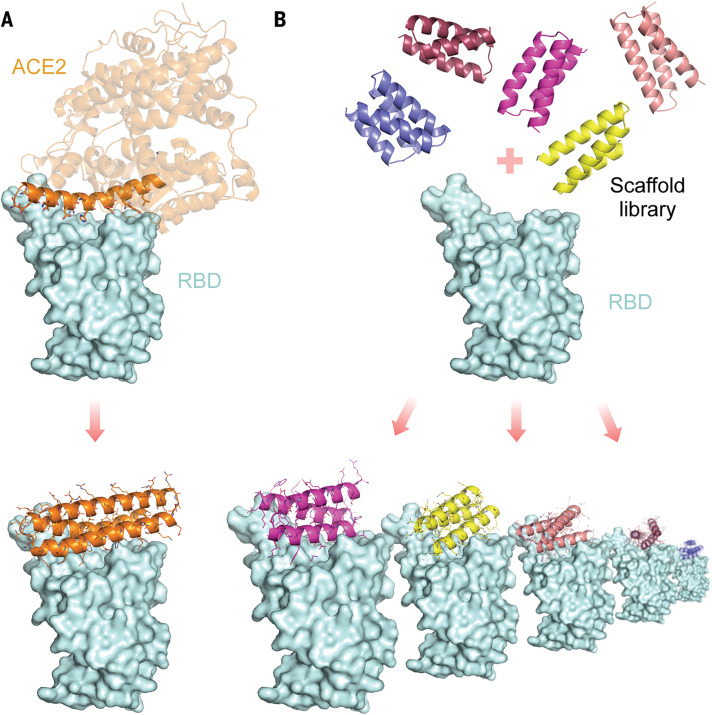
Overview of the computational design approaches. (**A**) Design of helical proteins incorporating ACE2 helix. (**B**) Large-scale de novo design of small helical scaffolds (top) followed by RIF docking to identify shape and chemically complementary binding modes.

We set out to design high-affinity protein minibinders to the SARS-CoV-2 spike receptor binding domain (RBD) that compete with ACE2 binding. We explored two strategies: First, we incorporated the α-helix from ACE2, which makes the majority of the interactions with the RBD into small designed proteins that make additional interactions with the RBD to attain higher affinity (Fig. 1A). Second, we designed binders completely from scratch, without relying on known RBD-binding interactions (Fig. 1B). An advantage of the second approach is that the range of possibilities for design is much larger, and so potentially a greater diversity of high-affinity binding modes can be identified. For the first approach, we used the Rosetta blueprint builder to generate miniproteins that incorporate the ACE2 helix (human ACE2 residues 23 to 46). For the second approach, we used rotamer interaction field (RIF) docking (*12*) with large in silico miniprotein libraries (*11*) followed by design to generate binders to distinct regions of the RBD surface surrounding the ACE2 binding site (Fig. 1 and fig. S1).

## Experimental characterization and optimization

Large pools of designed minibinders (supplementary materials, materials and methods), made by using the first and second approaches, were encoded in long oligonucleotides and screened for binding to fluorescently tagged RBD displayed on the surface of yeast cells. Deep sequencing identified three ACE2 helix scaffolded designs (“approach 1”), and 105 de novo interface designs (“approach 2”) that were enriched after fluorescence-activated cell sorting (FACS) for RBD binding. All three ACE2-scaffolded designs and 12 of the de novo designs were expressed in *Escherichia coli* and purified. One of the ACE2-scaffolded designs and 11 of the 12 de novo designs were soluble and bound RBD with affinities ranging from 100 nM to 2 μM in biolayer interferometry (BLI) experiments (figs. S2, A, C, and E; and S3). Affinity maturation of the ACE2-scaffolded design by means of polymerase chain reaction (PCR) mutagenesis led to a variant, AHB1, which bound RBD with an affinity of ~1 nM (fig. S4) and blocked binding of ACE2 to the RBD (fig. S5A), which is consistent with the design model, but had low thermostability (fig. S4, C and D). We generated 10 additional designs incorporating the binding helix hairpin of AHB1 and found that one bound the RBD and was thermostable (fig. S2, B, D, and F).

**Fig. 2:**
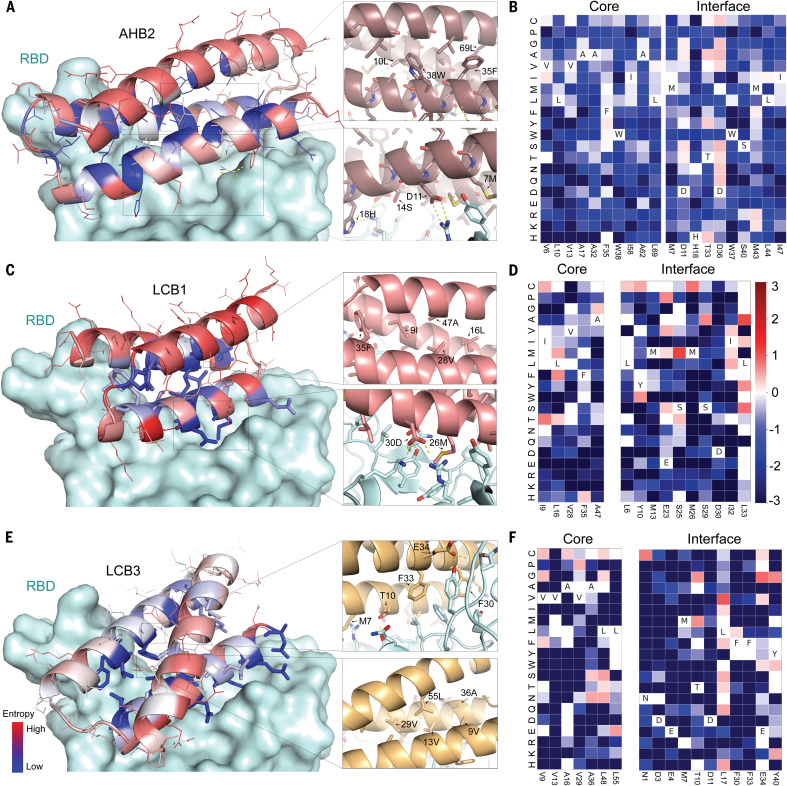
High-resolution sequence mapping of AHB2, LCB1, and LCB3 before sequence optimization. (**A**, **C**, and **E**) (Left) Designed binding proteins are colored by positional Shannon entropy from site saturation mutagenesis, with blue indicating positions of low entropy (conserved) and red those of high entropy (not conserved). (Right) Zoomed-in views of central regions of the design core and interface with the RBD. (**B**, **D**, and **F**) Heat maps representing RBD-binding enrichment values for single mutations in the design model core (left) and the designed interface (right). Substitutions that are heavily depleted are shown in blue, and beneficial mutations are shown in red. The depletion of most substitutions in both the binding site and the core suggest that the design models are largely correct, whereas the enriched substitutions suggest routes to improving affinity. Full SSM maps over all positions for AHB2 and all eight de novo designs are provided in figs. S6 and S7.

**Fig. 3:**
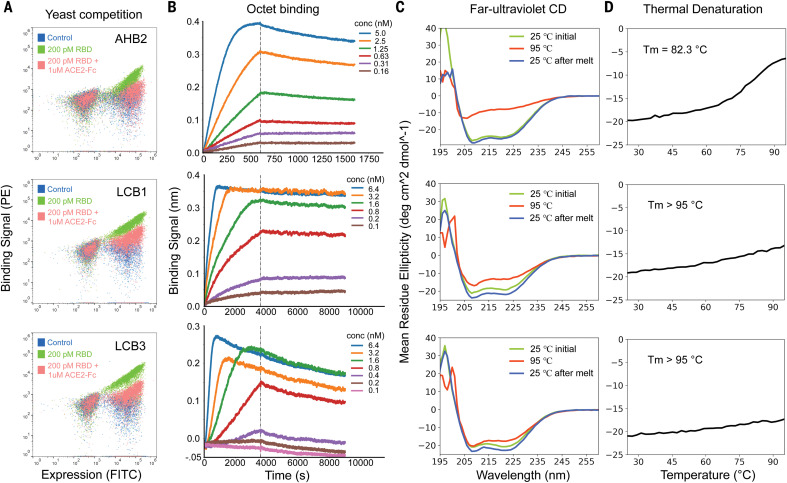
The optimized designs bind with high affinity to the RBD, compete with ACE2, and are thermostable. (**A**) ACE2 competes with the designs for binding to the RBD. Yeast cells displaying the indicated design were incubated with 200 pM RBD in the presence or absence of 1 μM ACE2, and RBD binding to cells (*y* axis) was monitored with flow cytometry. (**B**) Binding of purified miniproteins to the RBD monitored with BLI. For LCB1 and LCB3, dissociation constants (*K*_d_) could not be accurately estimated because of a lack of instrument sensitivity and long equilibration times below 200 pM. (**C**) Circular dichroism spectra at different temperatures and (**D**) CD signal at 222-nm wavelength, as a function of temperature. The fully de novo designs LCB1 and LCB3 are more stable than the ACE2 scaffolded helix design AHB2.

For 50 of the minibinders made by using approach 2, and the second-generation ACE2 helix scaffolded design, we generated site saturation mutagenesis libraries (SSMs) in which every residue in each design was substituted with each of the 20 amino acids one at a time. Deep sequencing before and after FACS sorting for RBD binding revealed that residues at the binding interface and protein core were largely conserved for 40 out of the 50 approach 2 minibinders and for the ACE2 helix scaffolded design (Fig. 2 and figs. S6 and S7). For most of these minibinders, a small number of substitutions were enriched in the FACS sorting; combinatorial libraries incorporating these substitutions were constructed for the ACE2-based design and the eight highest-affinity approach 2 designs and again screened for binding to the RBD at concentrations down to 20 pM. Each library converged on a small number of closely related sequences; one of these was selected for each design, AHB2 or LCB1-LCB8, and found to bind the RBD with high affinity on the yeast surface in a manner competed with by ACE2 (Fig. 3 and fig. S8).

AHB2 and LCB1-LCB8 were expressed and purified from *E. coli*, and binding to the RBD assessed with BLI. For seven of the designs, the dissociation constant (*K*_d_) values ranged from 1 to 20 nM (Fig. 3, fig. S8, and table S2), and for two (LCB1 and LCB3), the *K*_d_ values were below 1 nM, which is too strong to measure reliably with this technique (Fig. 3). On the surface of yeast cells, LCB1 and LCB3 showed binding signals at 5 pM of RBD after protease (trypsin and chymotrypsin) treatment (fig. S9). Circular dichroism spectra of the purified minibinders were consistent with the design models, and the melting temperatures for most were greater than 90°C (Fig. 3 and fig. S8). The designs retained full binding activity after 14 days at room temperature (fig. S10). AHB1 and -2 and LCB3 also bound to the SARS-CoV RBD (in addition to the SARS-CoV-2 RBD), but with lower affinity (fig. S11); we anticipate that the binding affinities achieved for SARS-CoV-2 could be readily obtained for other coronavirus spike proteins if these were directly targeted for design.

## Cryo–electron microscopy structure determination

**Fig. 4:**
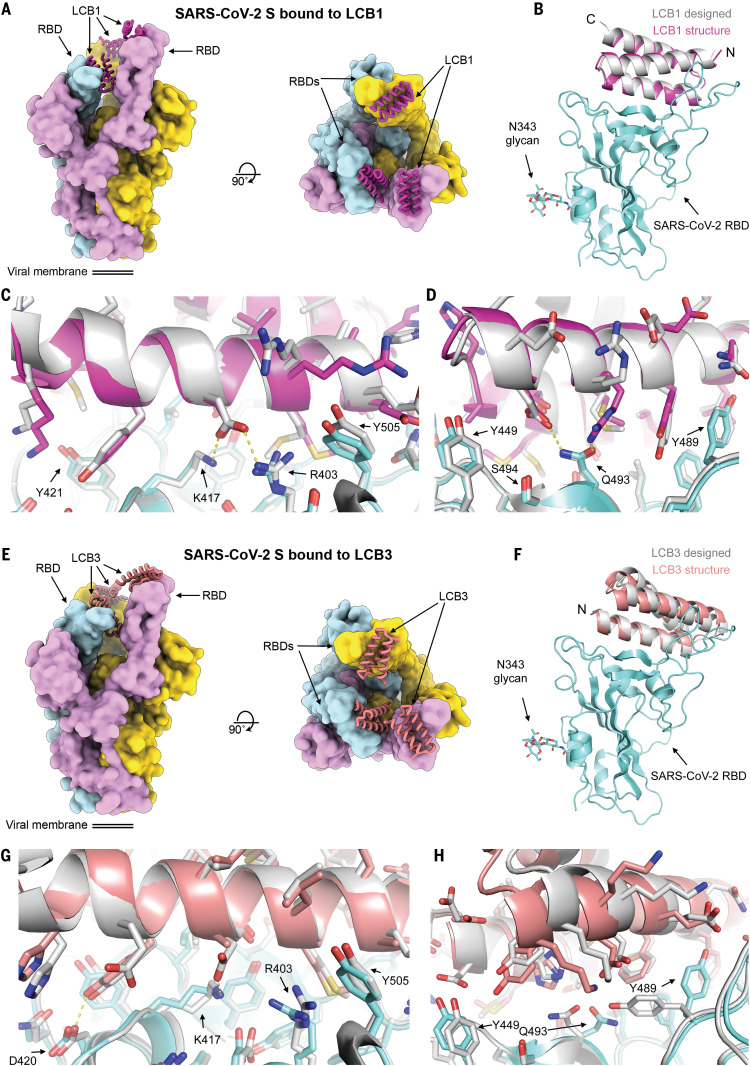
Cryo-EM characterization of the LCB1 and LCB3 minibinders in complex with SARS-CoV-2 spike protein. (**A**) Molecular surface representation of LCB1 bound to the SARS-CoV-2 spike ectodomain trimer viewed along two orthogonal axes (left, side view; right, top view) (**B**) Superimposition of the computational design model (silver) and refined cryo-EM structure (magenta) of LCB1 (using the map obtained through local refinement) bound to the RBD (cyan). (**C** and **D**) Zoomed-in views of computational model (silver) of LCB1/RBD complex overlaid on the cryo-EM structure (cyan for RBD and pink for LCB1), showing selected interacting side chains. (**E**) Molecular surface representation of LCB3 bound to the SARS-CoV-2 spike ectodomain trimer viewed from the side and top of the spike trimer. (**F**) Superimposition of the computational design model (silver) and refined cryo-EM structure (pink) of LCB3 (using the map obtained through local refinement) bound to the RBD (cyan). (**G** and **H**) Zoomed-in view of the interactions between LCB3 (pink) and the SARS-CoV-2 RBD (cyan), showing selected interacting side chains. In (A) and (E), each spike protomer is colored distinctly (cyan, pink, and yellow). For (B) and (F), the RBDs were superimposed to evaluate the binding pose deviations between designed models and refined structure of each minibinder.

We characterized the structures of LCB1 and LCB3 in complex with the SARS-CoV-2 spike ectodomain trimer by cryo–electron microscopy (cryo-EM) at 2.7 and 3.1 Å resolution, respectively, and found that the minibinders bind stoichiometrically to the three RBDs within the spike trimer (Fig. 4, A and E, and figs. S12 and S13). Although the spike predominantly harbored two open RBDs for both complexes, we identified a subset of particles with three RBDs open for the LCB3 complex (Fig. 4, A and E, and figs. S12 and S13). We improved the resolvability of the RBD/LCB1 and RBD/LCB3 densities by using focused classification and local refinement yielding maps at 3.1 and 3.5 Å resolution, which enabled visualization of the interactions formed by each minibinder with the RBD (Fig. 4, B and F, and figs. S12 and S13).

LCB1 and LCB3 dock with opposite orientations in the crevice formed by the RBD receptor-binding motif through extensive shape complementary interfaces with numerous electrostatic interactions mediated by two out of the three minibinder α-helices (Fig. 4, B to D and F to H). Similar to ACE2, the LCB1 and LCB3 binding sites are buried in the closed S conformational state and require opening of at least two RBDs to allow simultaneous recognition of the three binding sites (Fig. 4, A and E). Both LCB1 and LCB3 form multiple hydrogen bonds and salt bridges with the RBD with buried surface areas of ~1 000 and ~800 Å^2^, respectively (Fig. 4, C, D, G, and H), which is consistent with the subnanomolar affinities of these inhibitors. As designed, the binding sites for LCB1 and LCB3 overlap with that of ACE2 (fig. S14 and table S1) and hence should compete for binding to the RBD and inhibit viral attachment to the host cell surface.

Superimposition of the designed LCB1/RBD or LCB3/RBD models to the corresponding cryo-EM structures, using the RBD as reference, show that the overall binding modes closely match the design models with backbone Cα root mean square deviation of 1.27 and 1.9 Å for LCB1 and LCB3, respectively (Fig. 4, B and F), and that the primarily polar sidechain-sidechain interactions across the binding interfaces present in the computational design models are largely recapitulated in the corresponding cryo-EM structures (Fig. 4, C, D, G, and H). These data show that the computational design method can have quite high accuracy. The structure comparisons in Fig. 4, C, D, G, and H are to the original design models; the substitutions that increased binding affinity are quite subtle and have very little effect on backbone geometry.

## Virus neutralization

**Fig. 5:**
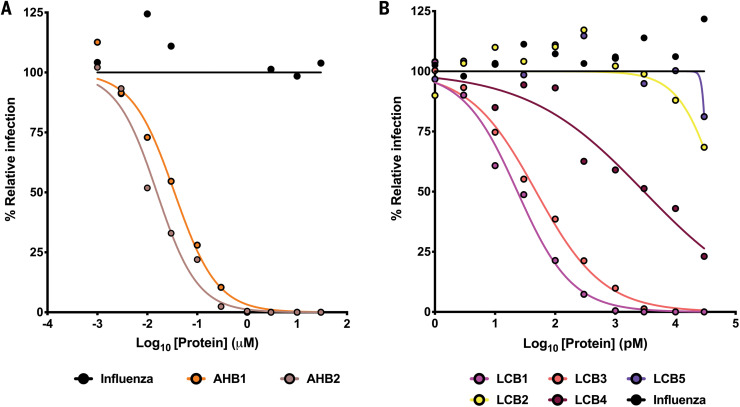
Neutralization of live virus by designed miniprotein inhibitors. (**A** and **B**) Neutralization activity of (A) AHB1 and AHB2 or (B) LCB1-5 were measured with a focus reduction neutralization test. Indicated concentrations of minibinders were incubated with 100 FFU of authentic SARS-CoV-2 and subsequently transferred onto Vero E6 monolayers. AHB1, AHB2, LCB1, and LCB3 potently neutralize SARS-CoV-2, with median effective concentration (EC_50_) values <50 nM (AHB1 and AHB2) or <50 pM (LCB1 and LCB3). Data are representative of two independent experiments, each performed in technical duplicate.

We investigated the capacity of AHB1, AHB2, and LCB1 to -5 to prevent the infection of cells by bona fide SARS-CoV-2. Varying concentrations of minibinders were incubated with 100 focus-forming units (FFU) of SARS-CoV-2 and then added to Vero E6 monolayers. AHB1 and AHB2 strongly neutralized SARS-CoV-2 (IC_50_ of 35 and 15.5 nM, respectively), whereas a control influenza minibinder showed no neutralization activity (Fig. 5A). Next, we tested the approach 2–designed minibinders LCB1 to LCB5. We observed even more potent neutralization of SARS-CoV-2 by LCB1 and LCB3 with IC_50_ values of 23.54 and 48.1 pM, respectively, within a factor of three of the most potent anti–SARS-CoV-2 monoclonal antibody described to date (13; at increased minibinder incubation volumes, IC_50_ values as low as 11 pM were obtained) (Fig. 5B). On a per mass basis, because of their very small size, the designs are sixfold more potent than the best monoclonal antibodies.

## Conclusions

The minibinders designed in this work have potential advantages over antibodies as potential therapeutics. Together, they span a range of binding modes, and in combination, viral mutational escape would be quite unlikely (figs. S1 and S14 and table S1). The retention of activity after extended time at elevated temperatures suggests that they would not require a temperature-controlled supply chain. The designs have only 5% the molecular weight of a full antibody molecule, and hence in an equal mass have 20-fold more potential neutralizing sites, increasing the potential efficacy of a locally administered drug. The cost of goods and the ability to scale to very high production should be lower for the much simpler miniproteins, which do not require expression in mammalian cells for proper folding, unlike antibodies. The small size and high stability should also make them amenable to formulation in a gel for nasal application and to direct delivery into the respiratory system through nebulization or as a dry powder. We will be exploring alternative routes of delivery in the months ahead as we seek to translate the high-potency neutralizing proteins into SARS-Cov2 therapeutics and prophylactics. Immunogenicity is a potential problem with any foreign molecule, but for previously characterized small de novo–designed proteins, little or no immune response has been observed (*11*, *14*), perhaps because the high solubility and stability together with the small size makes presentation on dendritic cells less likely.

Timing is critical in a pandemic outbreak; potent therapeutics are needed in as short a time as possible. We began to design minibinders in January 2020 on the basis of a Rosetta model of the SARS-CoV-2 spike structure and switched to the crystal structures once they became available (*4*, *15*–*17*). By the end of May 2020, we had identified very potent neutralizers of infectious virus; during this same time, a number of neutralizing monoclonal antibodies were identified. We believe that with continued development, the computational design approach can become much faster. First, as structure prediction methods continue to increase in accuracy, target models suitable for design could be generated within a day of determining the genome sequence of a new pathogen. Second, with continued improvement in computational design methods, it should be possible to streamline the workflow described here, which required screening of large sets of computational designs, followed by experimental optimization, to identify very-high-affinity binders. The very close agreement of the cryo-EM structures of LCB1 and LCB3 with the computational design models suggest that the main challenges to overcome are not in the de novo design of proteins with shape and chemical complementarity to the target surface, but in recognizing the best candidates and identifying a small number of affinity-increasing substitutions. The large amount of data collected in protein-interface design experiments such as those described here should inform the improvement of the detailed atomic models at the core of Rosetta design calculations, as well as complementary machine-learning approaches, to enable even faster in silico design of picomolar inhibitors such as LCB1 and LCB3. With continued methods development, we believe that it will become possible to generate ultrahigh-affinity, pathogen-neutralizing designs within weeks of obtaining a genome sequence. Preparing against unknown future pandemics is difficult, and such a capability could be an important component of a general response strategy.

## Supplementary Materials

science.sciencemag.org/content/370/6515/426/suppl/DC1 Materials and Methods Figs. S1 to S14 Tables S1 to S3 References (*18*–*39*)
